# The effect of poverty on developmental screening scores among infants

**DOI:** 10.1590/S1516-31802010000500007

**Published:** 2010-09-02

**Authors:** Giselle Souza de Paiva, Ana Cláudia Vasconcelos Martins de Souza Lima, Marilia de Carvalho Lima, Sophie Helena Eickmann

**Affiliations:** I MSc. Physiotherapist, Universidade Estadual de Ciências da Saúde de Alagoas (UNCISAL), Maceió, Alagoas, Brazil.; II OT, PhD. Associate professor, Department of Occupational Therapy, Universidade Federal de Pernambuco (UFPE), Recife, Pernambuco, Brazil.; III MD, PhD. Associate professor, Department of Maternal and Child Health, Universidade Federal de Pernambuco (UFPE), Recife, Pernambuco, Brazil.; IV MD, PhD. Associate professor, Department of Maternal and Child Health, Universidade Federal de Pernambuco (UFPE), Recife, Pernambuco, Brazil.

**Keywords:** Child development, Socioeconomic factors, Poverty, Primary health care, Infant, Desenvolvimento infantil, Fatores socioeconômicos, Pobreza, Atenção primária à saúde, Lactente

## Abstract

**CONTEXT AND OBJECTIVE:**

Child development is negatively influenced by multiple risk factors associated with poverty, thus indicating the importance of identifying the most vulnerable groups within populations that are apparently homogeneous regarding their state of socioeconomic deprivation. This study aimed to identify different levels of poverty in a population of low socioeconomic condition and to ascertain their influence on infants’ neuropsychomotor development.

**DESIGN AND SETTING:**

Cross-sectional study conducted at four Family Health Units in the Health District IV in the city of Recife, Brazil.

**METHODS:**

The sample comprised 136 infants aged 9 to 12 months, which represented 86% of all the infants in this age group, registered at the units studied. Socioeconomic status was assessed through a specific index and child development through the Bayley III screening test.

**RESULTS:**

Around 20% of the families were in the lowest quartile of the socioeconomic level index and these presented the highest frequency of infants with suspected delay in receptive communication. Maternal and paternal unemployment negatively influenced receptive communication and cognition, respectively. Not possessing a cell phone (a reflection of low socioeconomic status) was associated with worse cognitive performance and gross motricity. Male infants showed a higher frequency of suspected delay in receptive communication.

**CONCLUSIONS:**

Infants of more precarious socioeconomic status more frequently present suspected developmental delay. Development monitoring and intervention programs should be encouraged for this subgroup, thereby providing these children with a better chance of becoming productive citizens in the future.

## RESUMO

**CONTEXTO E OBJETIVO:**

O desenvolvimento infantil é influenciado negativamente por múltiplos fatores de risco associados à pobreza, tornando relevante a identificação de grupos mais vulneráveis numa população aparentemente homogênea quanto à sua condição de privação socioeconômica. O objetivo deste estudo foi identificar diferentes níveis de pobreza em uma população de baixa condição socioeconômica e verificar sua influência no desenvolvimento neuropsicomotor de lactentes.

**DESENHO E LOCAL DO ESTUDO:**

Estudo transversal realizado em quatro Unidades de Saúde da Família do Distrito Sanitário IV da cidade do Recife, Brasil.

**MÉTODOS:**

Amostra constou de 136 crianças entre 9 e 12 meses de vida, representando 86% dos lactentes cadastrados nesta faixa etária nas unidades estudadas. A condição socioeconômica foi avaliada segundo um índice de medição específico e o desenvolvimento através do teste de triagem da Bayley III.

**RESULTADOS:**

Cerca de 20% das famílias encontravam-se no quartil inferior do índice do nível socioeconômico, e apresentaram frequência mais elevada de crianças com suspeita de atraso na comunicação receptiva. O desemprego materno e paterno influenciou negativamente a comunicação receptiva e cognição, respectivamente. Não possuir telefone celular, refletindo o baixo nível socioeconômico, esteve associado a um pior desempenho cognitivo e da motricidade grossa. As crianças do sexo masculino apresentaram maior frequência de suspeita de atraso na comunicação receptiva.

**CONCLUSÕES:**

Lactentes com condições socioeconômicas mais precárias apresentam mais frequentemente suspeita de atraso no desenvolvimento. A vigilância ao desenvolvimento e programas de intervenção deve ser enfatizada para este subgrupo, proporcionando-lhes maior chance de se tornarem cidadãos produtivos no futuro.

## INTRODUCTION

With infant mortality rates on the decline over the last few decades, greater attention has been paid to observing biopsychosocial risk factors among children, thus emphasizing early detection of delayed neuropsychomotor development, especially in low socioeconomic groups.^1^

A recent study estimated that more than 200 million children under the age of five years may not achieve their full cognitive developmental potential due to poverty, precarious health and nutritional conditions and lack of environmental stimulation.^2^ Children in situations of poverty are exposed to multiple biological and environmental risk factors that have a cumulative and dynamic influence on neuropsychomotor development.^3-9^ During the first years of life, the impact of these factors becomes even more significant, since during this period there is rapid brain growth and intensive development of cognitive and motor skills. This is a time when the central nervous system is extremely vulnerable to environmental influences.^3^

A number of authors have studied the mechanisms through which poverty may influence infant development.^3,4,6-13^ These findings have suggested that delayed neuropsychomotor development is more serious and most probably occurs when children are obliged to live under conditions of extreme poverty for long periods of their lives.^7,13,14^ Poverty limits children’s access to stimulation and learning, due to the lack of available material resources.^4,6,7,11,13,15^ It also exposes them to stressful conditions, of both physical^4,11-14^ and psychosocial nature.^4,9,12,13,15,16^ There is evidence that these factors may lead to delays in cognitive, socioemotional and linguistic development among children.^2,4,5,9,10,13^

However, since poverty is such a complex theme, many of these issues have still not been well established. The majority of studies investigating this field have grouped individuals together in a broad category of low socioeconomic conditions, and have not taken into consideration the different levels of poverty and the possible concentration of risk factors at some of these levels.^10,14,17^

Recognizing that poverty is a more predominant feature in developing countries such as Brazil, and that the northeastern city of Recife portrays profound inequalities within its regional health districts, and consequently presents multiple risk factors for neuropsychomotor development, it would be of great importance to identify the groups most vulnerable to developmental delay. Through such findings, it might be possible to emphasize the importance of public policies that provide continuous and effective monitoring of infant development, and send any children at risk to referral centers as promptly as possible, thus favoring their future as productive citizens.

## OBJECTIVE

The aim of this study was to identify the different levels of poverty in a population of low socioeconomic level and to ascertain the influence of poverty on the neuropsychomotor development of infants attended within the Family Health Program.

## METHODS

### Study design and setting

A cross-sectional study with an analytical component was conducted in four Family Health Units located in Microregion 4.2 of Regional Health District IV in the city of Recife. The total population of this District is 271,200 inhabitants,^18^ and the population of Microregion 4.2 is 42,950 inhabitants. Microregion 4.2 was selected because, according to data from the Recife city authorities,^19^ it is predominantly made up of areas with low-income communities, and it is considered to be particularly precarious in terms of health determinants for both women and infants.

### Population and sample

All children aged between 9 and 12 months who were registered at the Family Health Units between February and August 2008 were considered eligible for the study. Those with severe neurological abnormalities (severe sensory disorders, cerebral palsy or mental deficiency) were excluded, leaving a total of 159 children. During data collection, 19 children (12%) either did not attend the evaluation even after being summoned for a second time, or their parents/caretakers refused to participate in the study. The study sample was therefore composed of 136 children.

### Socioeconomic status and biological condition of the children

Data were gathered between February and August 2008 by seven research assistants, who were all students from the Physiotherapy and Occupational Therapy courses at the Federal University of Pernambuco. The data gathering method consisted of interviews with either the mothers or the caregivers, at the Family Health Units, using a structured form with closed-end precoded questions.

The socioeconomic status of the family was assessed according to a measuring instrument that contained 13 items: parents’ schooling and occupations, number of people living in the home, paternal cohabitation, type of housing and ownership, number of people who slept in the house in relation to the number of beds available, the condition of the running water, sanitation, garbage collection, electricity, separation of the kitchen and ownership of household goods (refrigerator, television, cooker and radio). Each item received one point, and the sum of the points established the socioeconomic level of the family, with a possible range from 6 to 52 points. The present study used the instrument created by Alvarez et al.,^20^ which had been adapted to Brazilian realities by Issler and Giugliani.^17^ In this, the families are grouped in quartiles according to the final index obtained by the instrument. Thus, it may be considered that the lowest quartile of this instrument corresponds to the lowest socioeconomic level of the population.

Aditionally to the items of the instrument, other socioeconomic indicators were also gathered, such as: *per capita* family monthly income, possession of a DVD player, conventional and cell telephones, number of children under five years of age, and the age of the mother or caregiver. In relation to the characteristics of the child, the variables studied were: sex, age, duration of exclusive breastfeeding and any previous occurrence of hospitalization.

### Anthropometric assessment

The anthropometric assessment (weight, length and head circumference) was conducted by the principal investigator by means of standard equipment and techniques, as established by the World Health Organization (WHO).^21^ WHO standard references were adopted (WHO *Anthro* 2006, version 2.0), in order to assess the nutritional state, in terms of weight-for-age, length-for-age and head circumference-for-age expressed as mean z-scores.

### Assessment of neuropsychomotor development

Neuropsychomotor development screening was performed using the Bayley Scales of Infant and Toddler Development Screening Test, III Edition^22^ (Bayley III). This test was developed to identify the risk of developmental delays among children between the ages of 1 and 42 months, as well as to assist professionals in determining the need for further assessment of a broader nature. The test is subdivided into five subtests: Cognition, Receptive Communication, Expressive Communication, Fine Motor Skills and Gross Motor Skills. The points system of the subtests produces the scores, thus making it possible for the examiner to determine a cutoff point for each subtest administered in different age ranges. These cutoff points are used to determine into which category the infant may be placed: "Competent" (shows competence in tasks suitable for the age range); "Emerging" (reveals that abilities are still emerging, and is considered to be at risk of developmental delay); or "At Risk" (in need of further, more comprehensive evaluation in order to identify the developmental delay).^22^

Each test lasted approximately 15 to 20 minutes and followed the exact specifications of the application rules contained within the original manual of the Bayley III Screening Test. All data were registered on the appropriate report forms. Tests were performed at the Family Health Units by a duly trained researcher who was qualified in the field of infant development. In order to ensure quality control, inter-observer assessment was carried out by another author who is also a specialist in child development, and 9% of the tests were scored independently. Rater agreement (kappa index) of 0.63 was found for the Fine Motor Skills subtest, and it was greater than 0.90 for the remaining subtests.

### Data processing and analysis

The questionnaires were checked regularly for consistency, and data processing and analysis were performed using the Epi Info statistical software, version 6.04. Double data entry was used, with verification for consistency and validity.

Since undernutrition (< -2 z-scores) was only found in two and three children assessed using weight-for-age and length-for-age, respectively, the nutritional variables were grouped into two categories: well nourished (> -1 z-score) and at nutritional risk/undernourished (≤ -1 z-score). The Fine Motor and Expressive Communication subtests were excluded from the analysis because of the low numbers of children in the "Emerging" and "At risk" categories. This was also the reason for combining the "Emerging" and "At Risk" categories in the Gross Motor assessment.

Differences between the groups were assessed using the chi-square test with Yates correction and the Fisher exact test when indicated. The statistical significance level was taken to be P ≤ 0.05.

### Ethical issues

This study was approved by the Research Ethics Committee of the Agamenon Magalhães Hospital, in accordance with Resolution 196/96 of the National Commission for Research Ethics of the National Health Council. All the mothers/caregivers agreed to participate in the study by means of signing the written informed consent statement.

## RESULTS

The sample was composed of 136 infants, of which 57% were male. Two thirds (62%) had been exclusively breastfed for three months or more. The percentages of infants at nutritional risk/undernourished through weight-for-age and length-for-age were 7.5% and 22%, respectively, although of these, only two (1.5%) and three (2.2%) were malnourished, respectively.

The family socioeconomic status index ranged from 30 to 51 points, with a median value of 45 points (25^th^ to 75^th^ percentile range = 40-49). Most of the families were living in reasonable housing conditions and possessed several household goods. However, it was confirmed that the majority of this population was poor, such that around 78% of the sample received an income below the poverty line (≤ 0.5 minimum salary *per capita/* month) and 20% were in the lowest quartile of the socioeconomic status index.

In Table 1, it can be seen that most of the infants were in the "Competent" category, for all the developmental domains studied. However, in relation to receptive communication a high percentage of the infants (41%) was in the "Emerging" category, unlike the other domains, in which this percentage varied between 1 and 12%. Only two infants (1.5%) were placed in the "At Risk" category in the gross motor domain.

**Table 1.** Bayley III screening subtest scores among infants

Screening subtests	Competent		Emerging		At risk	
	n	%	n	%	n	%
Cognitive	119	87.5	17	12.5	0	0
Receptive communication	80	58.8	56	41.2	0	0
Expressive communication	131	96.3	5	3.7	0	0
Gross motor skills	122	89.7	12	8.8	2	1.5
Fine motor skills	135	99.3	1	0.7	0	0

Table 2 shows that the infants within the lowest quartile of the socioeconomic status index presented a higher percentage of suspected delay than did those in the other levels, with a statistically significant difference only in the receptive communication domain. The same trend was observed for the infants whose families had a lower *per capita* family income, although this difference was borderline (Table 3).

**Table 2.** Socioeconomic index and some of its indicators that were associated with Bayley III screening subtest scores among infants

Variables		Bayley III screening subtests								
	Total n (%)	Cognitive			Receptive communication			Gross motor		
		C	E	P	C	E	P	C	E	P
		n (%)	n (%)		n (%)	n (%)		n (%)	n (%)	
**Socioeconomic index**											
Lowest quartile	27 (19.9)	22 (81.5)	5 (18.5)	0.33*	11 (40.7)	16 (59.3) 40 (36.7)	0.05	23 (85.2)	4 (14.8)	0.48*
Higher quartiles	109 (80.1)	97 (89.0)	12 (11.0)		69 (63.3)			99 (90.8)	10 (9.2)	
**Family size**										
≤ 4	60 (44.1)	51 (85.0)	9 (15.0)	0.60	38 (63.3)	22 (36.7)	0.44	49 (81.7)	11 (18.3)	0.01
> 4	76 (55.9)	68 (89.5)	8 (10.5)		42 (55.3)	34 (44.7)		73 (96.1)	3 (3.9)	
**Head of family’s employment**											
Unemployed	72 (52.9)	58 (80.6)	14 (19.4)	0.02	36 (50.0)	36 (50.0)	0.04	62 (86.1)	10 (13.9)	0.24
Employed	64 (47.1)	61 (95.3)	3 (4.7)		44 (68.8)	20 (31.3)		60 (93.8)	4 (6.3)	
**House ownership**											
Borrowed	68 (50.0)	60 (88.2)	8 (11.8)	0.99	32 (47.1)	36 (52.9)	0.009	60 (88.2)	8 (11.8)	0.97
Rented/Owned	68 (50.0)	59 (86.8)	9 (13.2)		48 (70.6)	20 (29.4)		52 (89.7)	6 (10.3)	
**Toilet**											
None/latrine	14 (10.3)	9 (64.3)	5 (35.7)	0.02*	4 (28.6)	10 (71.4)	0.03	14 (100)	0	0.36*
Flush	122 (89.7)	110 (90.2)	12 (9.8)		76 (62.3)	46 (37.7)		108 (88.5)	14 (11.5)	
**Radio**											
No	34 (25.0)	26 (76.5)	8 (23.5)	0.03*	21 (61.8)	13 (38.2)	0.84	28 (82.4)	6 (17.6)	0.11*
Yes	102 (75.0)	93 (91.2)	9 (8.8)		59 (57.8)	43 (42.2)		94 (92.2)	8 (7.8)	
**Television**											
No	7 (5.1)	5 (71.4)	2 (28.6)	0.21*	2 (28.6)	5 (71.4)	0.12*	4 (57.1)	3 (42.9)	0.02*
Yes	129 (94.9)	114 (88.4)	15 (11.6)		78 (60.5)	51 (39.5)		118 (91.5)	11 (8.5)	

C = Competent; E = Emerging

* Fisher exact test.

**Table 3.** Socioeconomic and demographic conditions associated with Bayley III screening subtest scores among infants

Variables		Bayley III screening subtests								
	Total n (%)	Cognitive			Receptive communication			Gross motor		
		C	E	P	C	E	P	C	E	P
		n (%)	n (%)		n (%)	n (%)		n (%)	n (%)	
**Per capita family income (MS)** *											
≤ 0.50	91 (78.4)	81 (89.0)	10 (11.0)	1.00†	51 (56.0)	40 (44.0)	0.08	81 (89.0)	10 (11.0)	0.45
	> 0.50	25 (21.6)	23 (92.0)		2 (8.0)	19 (76.0)		6 (24.0)	24 (96.0)		1 (4.0)
**Maternal age (years)**											
≤ 19	32 (23.5)	26 (81.3)	6 (18.8)	0.23†	16 (50.0)	16 (50.0)	0.34	29 (90.6)	3 (9.4)	1.00†
≥ 20	104 (76.5)	93 (89.4)	11 (10.6)		64 (61.5)	40 (38.5)		93 (89.4)	11 (10.6)	
**Maternal schooling (years)**											
≤ 4	15 (11.0)	13 (86.7)	2 (13.3)	1.00†	7 (46.7)	8 (53.3)	0.46	15 (100)	0 (0.0)	0.36†
≥ 5	121 (89.0)	106 (87.6)	15 (12.4)		73 (60.3)	48 (39.7)		107 (88.4)	14 (11.6)	
**Paternal schooling (years)** ‡											
≤ 7	36 (37.1)	55 (90.2)	6 (9.8)	0.35†	17(47.2)	19 (52.8)	0.06	32 (88.9)	4 (11.1)	0.46†
≥ 8	61 (62.9)	30 (83.3)	6 (16.7)		42 (68.9)	19 (31.1)		57 (93.4)	4 (6.6)	
**Maternal employment**										
Housewife	112 (82.4)	96 (85.7)	16 (14.3)	0.31†	60 (53.6)	52 (46.4)	0.01	99 (88.4)	13 (11.6)	0.46†
Employed	24 (17.6)	23 (95.8)	1 (4.2)		20 (83.3)	4 (16.7)		23 (95.8)	1 (4.2)	
**Paternal employment** §											
Unemployed	41 (41.8)	32 (78.0)	9 (22.0)	0.03	21 (51.2)	20 (48.8)	0.13	37 (90.2)	4 (9.8)	0.72†
Employed	57 (58.2)	54 (94.7)	3 (5.3)		39 (68.4)	18 (31.6)		53 (93.0)	4 (7.0)	
**Children under five**										
> 1	97 (71.3)	35 (89.7)	4 (10.3)	0.78†	18 (46.2)	21 (53.8)	0.09	34 (87.2)	5 (12.8)	0.54†
1	39 (28.7)	84 (86.6)	13 (13.4)		62 (63.9)	35 (36.1)		88 (90.7)	9 (9.3)	
**Cell phone**											
No	26 (19.1)	19 (73.1)	7 (26.9)	0.02†	11 (42.3)	15 (57.7)	0.09	20 (76.9)	6 (23.1)	0.03†
Yes	110 (80.9)	101 (90.9)	10 (9.1)		69 (62.7)	41 (37.3)		102 (92.7)	8 (7.3)	

C = Competent; E = Emerging; MS = Minimum salary (1 MS = R$ 415.00)

* missing information in 20 cases

† Fisher exact test

‡ missing information in 39 cases

§ missing information in 38 cases.

From analysis on some of the indicators that make up the socioeconomic status index (Table 2), it can be seen that the number of people living in the home, the profession of the head of the family, the type of house ownership, type of sanitation and possession of radio and television had a significant influence over the development of the infants.

Table 3 presents correlations between infant development and other socioeconomic and demographic variables that do not form part of the socioeconomic status index. A tendency towards an association between paternal schooling and the development of receptive communication was identified, but the maternal schooling variable did not show any significant impact on the infant developmental domains studied. It can also be seen that maternal and paternal unemployment had negative impacts on the receptive communication and cognitive domains. In families with more than one child under the age of five, there was a tendency towards lower achievement in receptive communication.

In relation to biological and nutritional characteristics (Table 4), it was seen that there was a significantly higher frequency of male infants in the "Emerging" category (suspected delay), in the receptive communication domain. Infant development did not show any significant associations with exclusive breastfeeding, occurrences of previous hospitalization or nutritional indexes.

**Table 4.** Biological and nutritional characteristics of infants that were associated with Bayley III screening subtest scores among infants

Variables		Screening subtests of Bayley III								
		Total n (%)	Cognitive			Receptive communication			Gross motor		
	C		E	P	C	E	P	C	E	P
	n (%)		n (%)		n (%)	n (%)		n (%)	n (%)	
**Sex**											
Male	77 (56.6)	64 (83.1)	13 (16.9)	0.13	37 (48.1)	40 (51.9)	0.006	67 (87.0)	10 (13.0)	0.37
Female	59 (43.4)	55 (93.2)	4 (6.8)		43 (72.9)	16 (27.1)		55 (93.2)	4 (6.8)	
**Exclusive breastfeeding (months)**											
0 - 2	52 (38.2)	46 (88.5)	6 (11.5)	1.00	28 (53.8)	24 (46.2)	0.45	46 (88.5)	6 (11.5)	0.93
≥ 3	84 (61.8)	73 (86.9)	11 (13.1)		52 (61.9)	32 (38.1)		76 (90.5)	8 (9.5)	
**Hospitalization**											
Yes	33 (24.3)	31 (93.9)	2 (6.1)	0.36*	22 (66.7)	11 (33.3)	0.40	30 (90.9)	3 (9.1)	1.00*
No	103 (75.7)	89 (85.6)	15 (14.4)		58 (56.3)	45 (43.7)		92 (89.3)	11 (10.7)	
**Length-for-age (z-score)** †											
< -1	30 (22.4)	24 (80.0)	6 (20.0)	0.21*	16 (53.3)	14 (46.7)	0.62	29 (96.7)	1 (3.3)	0.19*
≥ -1	104 (77.6)	93 (89.4)	11 (10.6)		63 (60.6)	41 (39.4)		91 (87.5)	13 (12.5)	
**Weight-for-age (z-score)**											
< -1	10 (7.5)	8 (80.0)	2 (20.0)	0.61*	4 (40.0)	6 (60.0)	0.32*	10 (100)	0	0.60*
≥ -1	124 (92.5)	109 (87.9)	15 (12.1)		75 (60.5)	49 (39.5)		110 (88.7)	14 (11.3)	
**Head circumference-for-age (z-score)** ‡											
≤ 0	39 (28.9)	32 (82.1)	7 (17.9)	0.26*	19 (48.7)	20 (51.3)	0.16	37 (94.9)	2 (5.1)	0.35*
> 0	96 (71.1)	86 (89.6)	10 (10.4)		61 (63.5)	35 (36.5)		85(88.5)	11 (11.5)	

C = Competent; E = Emerging

* Fisher exact test

† missing information in 2 cases

‡ missing information in 1 case.

## DISCUSSION

The results from the present study suggest that infants between the ages of 9 and 12 months living under conditions of lower socioeconomic status showed a risk of delayed development when assessed using the Bayley III screening test, especially in relation to the development of receptive communication. Within the context of poverty, the unfavorable socioeconomic conditions expressed through maternal and paternal unemployment and the unavailability of household goods (radio, cell phone and television) had a negative impact on different subtests of this scale.

Because of the dynamic, multifactorial nature of poverty and infant development, attempting to compare the results from studies that have illustrated an association between these two phenomena proves to be a complex matter. In addition, these studies have also used different methods, not only to assess socioeconomic status^3,4,9,10^ but also to evaluate infant development.^3,10,23,24^

In the present study, the instrument for measuring socioeconomic status had the advantage of not including the *per capita* family income. Although this parameter has been used by several authors,^4,10,24,25^ accurate figures for this are somewhat difficult to obtain, especially in communities with low socioeconomic status, thus rendering data from the literature conflicting and hard to interpret.^7,9^

Moreover, it is important to highlight that a relationship between monthly *per capita* family income, which was specifically investigated among the participants in this study, and socioeconomic status analyzed using the instrument was observed. This means that the families in the lowest quartile of this index had in fact lower monthly *per capita* income, thus confirming the coherence between the index used and the purchasing power of the population, as revealed by the family income (data not shown).

There is substantial evidence in the literature concerning the short^3,4^ and long-term^2,8,10,12^ negative impacts of poverty on infant development during the first years of life. The present study showed that the most negatively affected developmental domain among infants from families with low socioeconomic status was receptive communication. The same tendency was also observed among families with low *per capita* incomes. These results corroborate those of Najman et al.,^10^ in a cohort study to evaluate the effects of low socioeconomic status on cognitive and emotional development. The group of mothers living under conditions of poverty during pregnancy was twice as likely to have children who, at five years of age, would present delayed verbal comprehension, even after adjustment for other socioeconomic variables.

The mechanism through which the association between poverty and infant development occurs has still not been well established in the literature. With regard to language, several studies have indicated that families with higher socioeconomic status tend to read more to their children,^7,9^ engage their children in richer discourse and provide more complex verbal strategies.^26^ Deprived of these stimuli, children from families of low socioeconomic status run a greater risk of delayed language development.^7,9^

The present study found a significant association between the type of house occupancy and possession of household goods (radio, cell phone and television) and different aspects of infant development. Lack of these material goods reflected families’ lower socioeconomic status,^4,14^ thus having a negative impact on children’s overall development. Possession of household goods, as an expression of socioeconomic status, has also been cited by other authors.^4,15^ Similarly, living in houses with poor sanitation, which had a negative impact on different domains of infant development within the present study, is a common characteristic of the most underprivileged housing areas and of the populations with the least community representation.^4^

Within this context, some authors have confirmed that the relationship between socioeconomic status and children’s cognitive and linguistic acquisition is permeated by stimulation from within the home and is associated with the degree of agglomeration within the household and the number of siblings living in the home.^7,9,16,25^ The findings from the present study showed a tendency towards delayed receptive communication when there was more than one child under the age of five years in the family. This effect may be related to the fact that parents are less available to provide their children with the due attention, thus reducing the opportunities for stimulation.^7,11^

Hence, it was observed that infants living in a family environment surrounded by large numbers of people achieved better performances in the gross motor skill domain. The greater adult/child proportion may have led to closer contact with a reference individual, thereby enabling more appropriate stimulation with positive repercussions on neuropsychomotor development.

The positive role of environmental stimulation in relation to infant development has been well documented in the literature. Experiments both with animals and with children have demonstrated that secure attachment and an enriched environment for development are protective factors.^9,15,27-30^ By means of a psychosocial stimulation program aimed at mothers, Eickmann et al.^15^ recorded a significant increase in the mental and motor development indexes, as assessed using Bayley II at 18 months, in the group that received the intervention, compared with the control group.

With regard to maternal employment, it was seen that this was associated with higher frequency of suspected delayed receptive communication development. Some authors have cited that economic instability at home, resulting from unemployment and a reduction in family income, constitutes an important risk factor whereby parents become more punitive and less communicative and responsive to the needs of the child,^13,27^ thus compromising linguistic stimulation.

Moreover, low income also prevents any access to the means for recreation and learning, which are considered to be materials for cognitive stimulation (books, newspapers, magazines and toys).^7^ Therefore, these factors may also be related to cognitive development, which is in accordance with the results of this study, in which paternal unemployment presented a negative impact on the cognitive domain.

Although there is evidence in the literature that good maternal schooling has positive repercussions on infant development,^9,25,27^ this characteristic was not found within the study population, probably due to the small sample size. On the other hand, there was an association between paternal schooling and receptive communication performance (borderline statistical significance, P = 0.06), which could be related to higher income and to stimulation within the home.

In relation to the biological and nutritional variables studied, it was observed that male infants obtained lower results in the domain of receptive communication than did females, which agrees with the findings of other authors.^3,4,28^ There is evidence that male children have lower cognitive performance during infancy,^4^ and this domain is considerably related to language. However, the mechanisms through which this association occurs are still not well established.

Although there is evidence that biological and environmental factors influence infant development in an interactive, cumulative manner, the environmental impact has featured heavily in the literature^3,4,7-15,24-31^ over the last decade. In a cohort study on 12 month-old infants in northeastern Brazil, Lima et al.^4^ found that environmental risk factors relating to poverty had a greater impact on infant development than did biological risk factors.

With regard to the instrument used in the present study for evaluating development, as far as we know, this was the first study to use the Bayley III screening test to evaluate the development of infants of low socioeconomic status. This test is a recently developed instrument, and it can be applied quickly and cheaply. Its validity, accuracy and trustworthiness have been tested on both normal and high-risk children in the United States.^22^

However, although the data were gathered as accurately as possible, including the care taken to evaluate the inter-examiner agreement, a discrepancy was encountered in the domain of receptive communication in relation to the other domains studied. While the proportion of the infants in the "Emerging" category was 41% for the receptive communication domain, this percentage was lower in the other domains, ranging from 1 to 12%. The literature indicates a higher frequency of false-positive results in screening tests^32^ than seen in the present study, in which this characteristic was only encountered in the domain of receptive communication. Thus, the current findings lead to a number of questions, since no reports of similar results are available in the literature, in studies that use screening tests for infant development.

It may be that these findings relate to certain recently undertaken adaptations to the instrument in question. Although the Bayley III screening test uses items from the Bayley III evaluation scale, which is considered to be the gold standard in this field, the evaluation of the scores is carried out in a different manner, thereby establishing cutoff points and categories. According to Drachler,^33^ the use of cutoff points in screening scales makes information gathering more difficult with regard to the variability of infant development, whereas this does not occur in evaluation scales with continuous indexes.

It is possible to correlate the high levels of suspected delay in receptive communication with the difficulty in accomplishing some of the items that was shown by some infants, thus making the examiner’s scoring subjective and leading to a lower score in this developmental domain. One example of this is item 7, "Responds to name", in which, in order to score, the child is required to turn its head on the two occasions when its name is spoken, but not in response to an unfamiliar name. What may be observed here is that the child, out of curiosity, generally turns its head in response to any name called out by the examiner, thus receiving a score of "zero" for this item. Similar observations were made in item 9, "Recognizes two familiar words". Thus, failure to accomplish items such as these may lead to a lower overall score in this developmental domain.

Furthermore, it is important to highlight that the present study also observed items that were easily performed by infants within the age range of this study, in relation to the scales of expressive communication and fine and gross motor skills. This may have led to higher competence scores. Thus, greater tolerance was confirmed in the cutoff points established by the instrument in these domains of infant development, thereby rendering it even more difficult for a child to be considered as "Emerging", since it would have to fail in a high number of items that were easy for that particular age range.

One possible limitation to the present study is the number of families who did not attend the evaluation or who refused to participate in the study (12%), even after the second call. Melo et al.^34^ identified from observation of healthcare workers that mothers take their children for pediatric monitoring at Family Health Units only when they are suffering from some health problem, which may explain the absence of some of the families that was seen during the present study.

Future longitudinal studies should analyze other issues that may influence infant development, such as the quality of the home environmental stimulation, maternal mental health, family stress and resilience traits within the population.

## FINAL CONSIDERATIONS

The population studied, which was predominantly poor in terms of *per capita* family income, exhibited strata of different characteristics that caused an impact on neuropsychomotor development in a number of different manners. The instrument used to evaluate socioeconomic status proved to be useful in identifying these strata. These findings reinforce the multifactorial nature of infant development and indicate the importance of providing early, continuous monitoring, especially among underprivileged populations.

Considering the discrepancy observed in the results from evaluating neuropsychomotor development performed using the Bayley III screening test, further studies should be carried out in an attempt to provide a better evaluation of this instrument.

## CONCLUSIONS

Infants of more precarious socioeconomic status more frequently present suspected developmental delay. Development monitoring and intervention programs should be encouraged for this subgroup, thereby providing these children with a better chance of becoming productive citizens in the future.
